# Effect of temperature on anisotropic bending elasticity of dsRNA: an all-atom molecular dynamics simulation†

**DOI:** 10.1039/d4ra02354d

**Published:** 2024-05-28

**Authors:** Xianghong Wang, Tingting Huang, Liyun Li, Yanliang Xu

**Affiliations:** a School of Sino-German Engineering, Shanghai Technical Institute of Electronics and Information Shanghai 201411 China wangxianghong@stiei.edu.cn; b Department of Physics, Wenzhou University Wenzhou 325035 China

## Abstract

Employing all-atom molecular dynamics simulations, we examined the temperature-dependent behavior of bending elasticity in double-stranded RNA (dsRNA). Specifically, we focused on the bending persistence length and its constituent components, namely, the tilt and roll stiffness. Our results revealed a near-linear decrease in these stiffness components as a function of temperature, thereby highlighting the increased flexibility of dsRNA at elevated temperatures. Furthermore, our data revealed a significant anisotropy in dsRNA bending elasticity, which diminished with increasing temperature, attributable to marked disparities in tilt and roll stiffness components. We delineated the underlying biophysical mechanisms and corroborated our findings with extant literature. These observations offer salient implications for advancing our understanding of nucleic acid elasticity, and are pertinent to potential medical applications.

## Abstract 2

The tilt stiffness *A*_1_ and roll stiffness *A*_2_ decrease with the increasing temperature.

## Graphical abstract

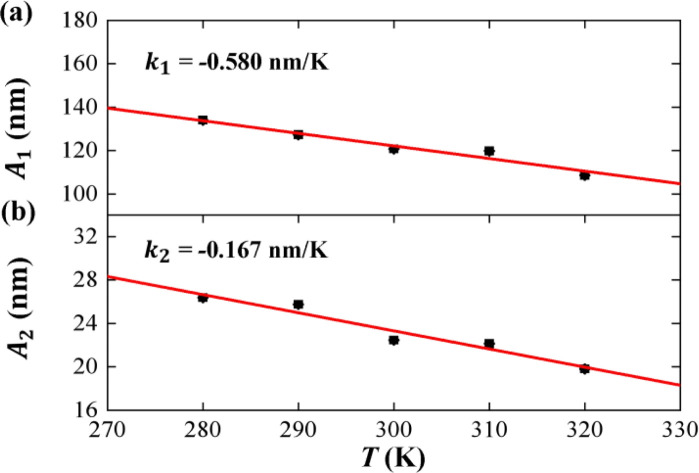

## 1 Introduction

RNAs are pivotal in various biological processes, including but not limited to the encapsulation of double-stranded RNA (dsRNA) viral genomes into capsids and structural rearrangements of ribosomes during translation.^1–3^ Within these biological contexts, RNA undergoes diverse mechanical deformations in response to alterations in physiological conditions such as stretching, bending, and twisting. Concurrently, dsRNA has garnered attention as a promising nanomaterial for biological and nanomedical applications.^3^ Bend elasticity is a fundamental mechanical property of nucleic acids and is instrumental in shaping their three-dimensional structures under physiological conditions.^4^ Accordingly, elucidating the impact of environmental variables such as ionic strength and temperature on dsRNA bend elasticity is crucial.

Traditionally, bending elasticity is quantified either *via* the bending persistence length or bending stiffness. For dsDNA, the bending persistence length is approximately 50 nm under standard physiological conditions, as described by the wormlike chain (WLC) model.^5,6^ Notably, the bending elasticity of dsDNA is sensitive to external conditions, including ionic concentration^7–11^ and temperature.^12–21^ Temperature elevations typically manifest as reductions in the bending persistence length of dsDNA, which is attributable to thermal fluctuations within the molecular milieu.^22^ For examples, the experiments suggested that the bending persistence length decreased as the temperature was increased,^17^ which have been reproduced in Monte Carlo (MC) and all-atom molecular dynamics (MD) simulations based on various models.^19,20,23^

For dsRNA, both experimental and computational data suggest a bending persistence length of approximately 60 nm under normal physiological conditions, surpassing that of dsDNA.^24–26^ For examples, the magnetic tweezer (MT) experiments reported that the dsRNA possesses the bending persistence lengths of *l*_B_ = 60 ± 1 nm,^24^*l*_B_ = 57 ± 2 nm (ref. 25) and *l*_B_ = ∼61 nm.^26^ The all-atom MD data reproduced that the dsRNA have the bending persistence length of *l*_B_ = 69 ± 4 nm,^27^*l*_B_ = 66.3 nm (ref. 25) and *l*_B_ = 66.99 ± 1.38 nm.^28^ The observed disparities between the MT and MD data can be attributed to variations in experimental conditions, such as ionic strength and sequence composition. The influence of sequence composition on elasticity mainly refers to the length and order of base pairs. Generally, the sequence length used in experiments is several thousand bp, while the sequence length used in all-atom simulations is several tens of bp due to the limitations of computational performance. It is noteworthy that the bending persistence length of dsRNA diminishes with an increase in ion concentration.^26^ Nevertheless, the impact of temperature on the bending persistence length of dsRNA remains an open avenue for investigation.

In the extant Marko and Siggia (MS) elastic model, bending elasticity is partitioned into two orthogonal modes, encapsulated by the tilt and roll angles.^29^ This partitioning underpins the observed anisotropy in bending rigidities, manifesting as differential flexibility along the major and minor grooves of nucleic acid helices.^30^ The MS model, extensively employed in dsDNA studies, accounts for the observed bending anisotropies and associated twist–bend couplings.^31–35^ Previous research cites tilt stiffness values of approximately 100 nm and roll stiffness values of approximately 39 nm for dsDNA at room temperature.^31,32,36,37^ The inherent asymmetry between these values correlates with the observed molecular geometry of the DNA.^36^ Such anisotropic behavior has also been observed in dsRNA, albeit with sequence-dependent variations in the extent of anisotropy.^38^ Recently, Dohnalová *et al.* used single-molecule MT measurements to determine the temperature-dependence of the dsRNA twist and observed that dsRNA unwinds with increasing temperature, which was correctly predicted by the all-atom MD simulations.^39^ Dabin *et al.* investigated the thermal denaturation of dsRNA using the atomistic simulations by varying the temperature in a wide range, in which the sequences and force fields were considered.^40^

In the present study, we employed all-atom molecular dynamics simulations of a representative dsRNA sequence to scrutinize the temperature-dependent properties of bending elasticity. Our study concentrates on the temperature effects on the bending persistence length, tilt, and roll stiffness, as well as the bending anisotropy, guided by the WLC and MS models. Section 2 outlines the simulation methodology, Section 3 discusses temperature-dependent findings, and Section 4 concludes.

## 2 Simulation models and method

### 2.1 All-atom MD simulation

**Fig. 1:**
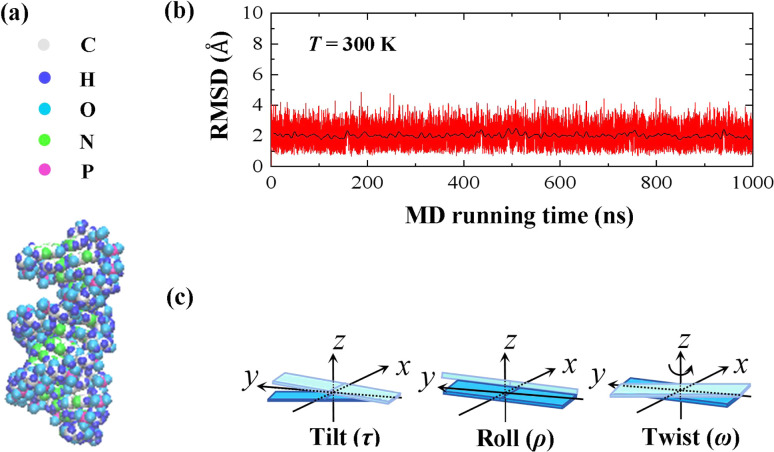
(a) Representative diagrams for the molecular structure of 16 bps dsRNA with 5′-GCGC AAUG GAGU ACGC-3′ sequence. (b) Root mean square deviation (RMSD) curves of the 10 bases fragment in the center of the dsRNA at *T* = 300 K, where the black line indicates the average value of the relevant parameter every 2 ns, which reached equilibrium after 100 ns. (c) Sketch diagrams for the tilt angle, roll angle, and twist angle on the molecular structure.

In the current simulations, we selected the initial A-type dsRNA with a 16 bp sequence of 5′-GCGC AAUG GAGU ACGC-3′, which has been used in the previous works.^41,42^ Our all-atom MD simulations are similar to those in previous studies,^21,43,44^ and we only briefly describe the content involved in the current simulations. We constructed the initial structure file of the RNA using the UCSF Chimera 1.15 software application,^45^ as shown in Fig. 1(a), where there are approximately 1026 atoms in the 16 bps sequence. The dsRNA sequence was immersed in an 8 × 8 × 8 nm^3^ simulation box under periodic boundary conditions. Subsequently, NaCl^46^ and water molecules were added corresponding to 100 mM salt concentration, where the TIP3P model^47^ was used to define the water structure. We added Na^+^ and Cl^−^ ions using by Joung and Cheatham's ion model^48^ to the environment to maintain a constant concentration. Because nucleic acids are negatively charged, additional 30 Na^+^ ions are dumped into the solution, which neutralizes the negative charge in the solution environment.

Our MD simulations were performed using the Gromacs 4.6 software with the Amber OL3 force fields.^49–53^ Before the MD simulations, the system needs to be pre-equilibrated to ensure that the MD simulation is performed in an isothermal isobaric environment.^54,55^ We started with an energy minimization system with a restraining force and subsequently an NVT and NPT simulations. In NVT, the system is warmed up to the desired temperature *T* by the *V*-rescale method.^54,55^ Specifically, this scheme uses the velocity-rescaling method with random terms for temperature coupling to produce the NVT canonical ensemble, retaining the Berendsen thermostat's advantages, which have the first-order decay of the temperature deviation and the absence of oscillations.^54,55^ After the system was preheated to the desired temperature *T*, the system pressure was adjusted to 1 atm using the NPT simulation. When these pre-equilibration processes were completed, and a 1000 ns MD simulation was performed at a fixed temperature *T* without the restraining force. We adjusted the temperature *T* from 280 K to 320 K, with a step of Δ*T* = 10 K, where the temperature regulation was similar to those of all-atom MD simulations with Amber force fields.^16,20,21,56,57^ We checked the validity of the simulation by calculating the root mean square deviation (RMSD) of the system. An example is shown in the RMSD plots for 16 bps dsRNA in Fig. 1(b). The RMSD indicates the extent of structural changes in the dsRNA molecule as follows:1
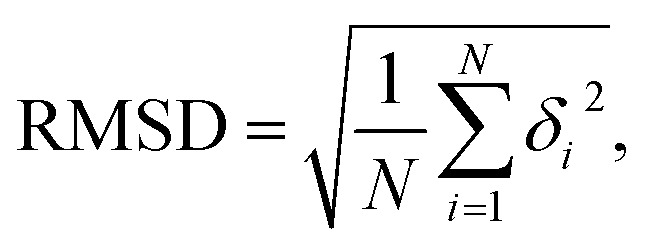
where *δ*_*i*_ is the displacement of the *i* atom from moment 0 to moment *t* with a time step of Δ*t* = 2 ps. We take each 2 ns as the average value and draw the black line in the middle as shown in Fig. 1(b). We also provided more RMSD plots at various temperatures in Fig. S1 of ESI.† These RMSD data indicated that these systems with various temperatures reach to the equilibrium states after 100 ns, respectively. In all simulations, elasticity data from 100 ns to 1000 ns were used for the subsequent statistical analysis. To avoid end effects, we removed 3 bps from each end of the sequence in all data analysis and only selected the central 10 bps to analyze the overall elastic properties of dsRNA.

### 2.2 WLC and MS models

In this study, we focused on the elastic parameters of the MS model and WLC models. In WLC model, the bend elasticity can be described as follows^9,58–60^2
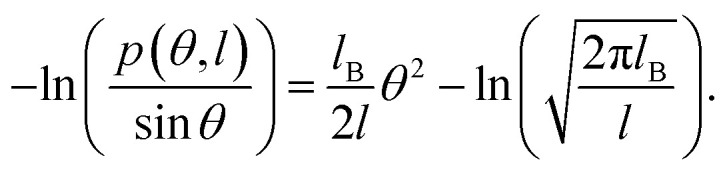
Here, *l*_B_ is the bending persistence length, *p*(*θ*, *l*) is the probability distribution; *θ* is the bending angle formed by a dsRNA spanning 6 bps;^61^*l* is a constant representing the average 6 bps profile length. The *l*_B_ value was obtained by fitting the quadratic function to eqn (2) using the MD simulation data. Here, we note that by removing several bps from each helix end in the data analysis, the short nucleic acid sequence can also be described by WLC model with the bending persistence length using eqn (2).^62^

The MS model was originally proposed by Marko and Siggia,^29^ where three angles *Ω*_1_, *Ω*_2_ and *Ω*_3_ was used to describe the bend and twist elasticities of DNA in recent years.^31,34,35,63^ These three rotational angles, *Ω*_1_, *Ω*_2_ and *Ω*_3_, are related to the tilt, roll and twist in the rigid base-pair,^37^ as shown in Fig. 1(c). In the MS model, by considering the symmetry and lowest order of Ω, one can express the energy functional as follows^29^3
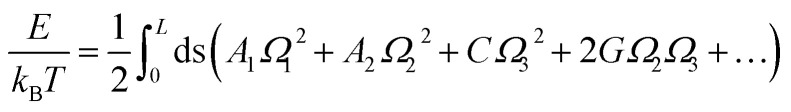
where *k*_B_ is the Boltzmann constant, *T* is the temperature and the dots denote the higher-order terms. The *A*_1_ and *A*_2_ are two bending stiffnesses, *C* is the torsional stiffness and *G* is the twist–bend coupling constant. These elastic parameters have length dimensions; thus *A*_1_, *A*_2_ and *C* also denote the corresponding persistence lengths. The MS model can be reduced to the twistable worm-like chain (TWLC) model by neglecting the twist–bend coupling *G*, and taking the energy functional as follows^32^4
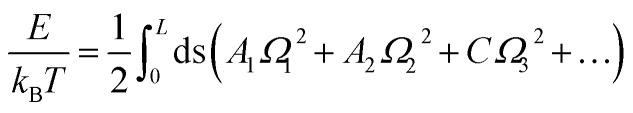


One can further reduce eqn (4) into WLC model by ignoring *Ω*_3_.

In the MS model, *A*_1_, *A*_2_, *C* and *G* are related to the elastic moduli *K*_*ττ*_, *K*_*ρρ*_, *K*_*ωω*_ and *K*_*ρω*_ multiplied by *L*_0_ in unit of *k*_B_*T*, where *L*_0_ is the contour length of dsRNA. According to the random thermal fluctuation, the elastic modulus **K** can be expressed as^22^5
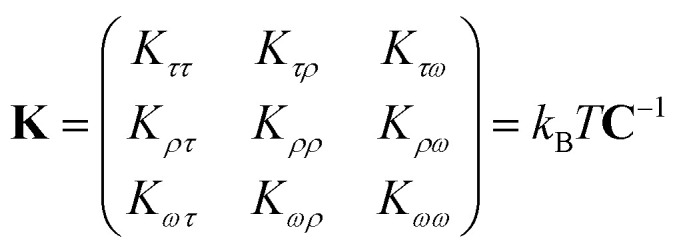
Here, matrix **C** is a covariance matrix with the elements

6 cov(*i*,*j*) = *c*_*ij*_*σ*_*i*_*σ*_*j*_

where *c*_*ij*_ is the Pearson coefficient of *i* and *j*, *σ*_*i*_ and *σ*_*j*_ are the standard deviations of *i* and *j*. The *i* and *j* denote the cumulative tilt *τ*, roll *ρ* and twist *ω*, which are MD-associated parameters in the simulations.

Generally, three angular parameters, tilt, roll and twist, can be used to describe the bending and twisting elasticities of dsRNA, as well as its coupling, in the rigid base-pair (RBP) model, as shown in eqn (5). Here, we try to describe the relationship between MS, TWLC and WLC models, starting from the RBP model. In MS model, only the coupling between the roll and twist, is considered, since the tilt is weakly coupled to roll and twist.^29^ However, the TWLC model further ignore the coupling between roll and twist angles.^32^ In WLC model, it only considers the tilt and roll angles by ignoring the twist angle, which can be used to describe the bending elasticities. Such approximation of the WLC model makes the MS model closer to the experiments.

## 3 Results and discussion

**Fig. 2:**
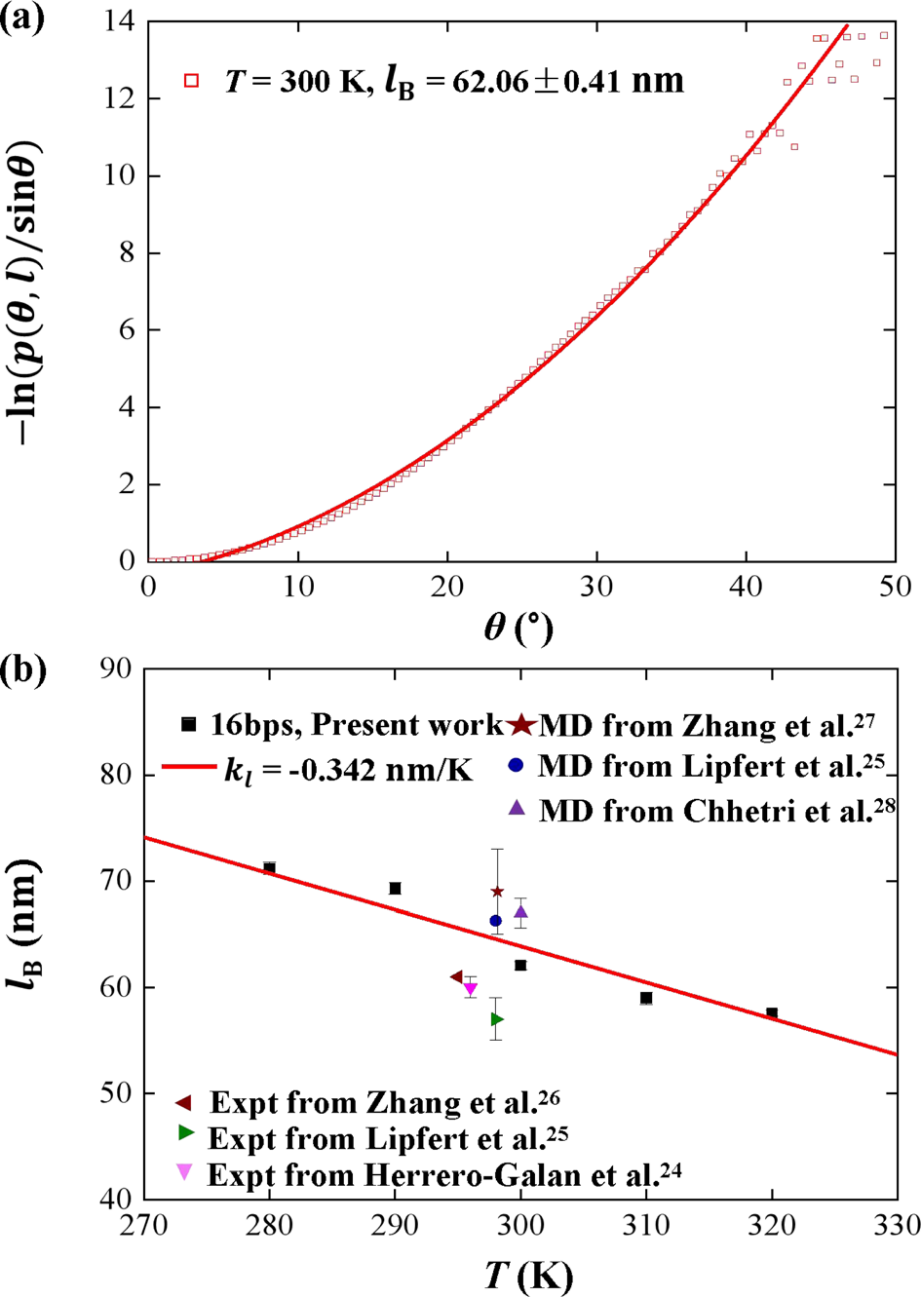
(a) The relationship between 
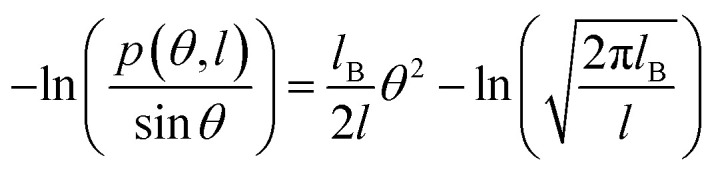
 and bending angle *θ* at *T* = 300 K. The bending angle *θ* is formed by six consecutive base pairs on each of the 10 base segments at the center of the dsRNA. (b) Temperature dependence of bending persistence length of dsRNA. The line is a fitting result with a slope of *k*_*l*_ = −0.342 nm K^−1^. The available data were also inserted for convenient comparison.

**Fig. 3:**
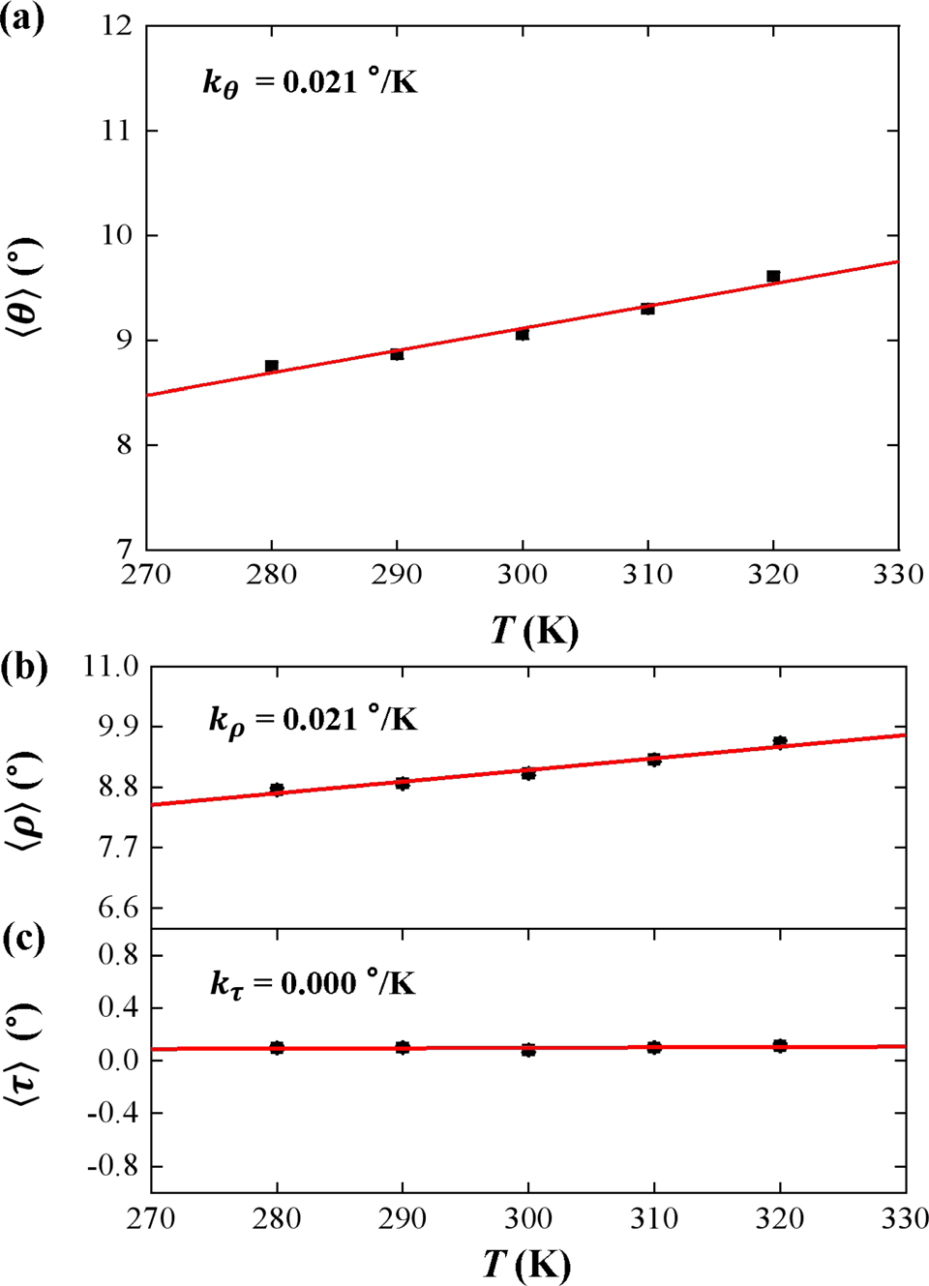
(a) The temperature dependent bending angle *θ*. The line is a fitting result with a slope of *k*_*θ*_ = 0.021° K^−1^. (b) The temperature dependent roll angle *ρ*. The lines are fitting results with slope of *k*_*ρ*_ = 0.021° K^−1^. (c) The temperature dependent tilt angle *τ*. The lines are fitting results with slope of *k*_*τ*_ = 0.000° K^−1^.

**Fig. 4:**
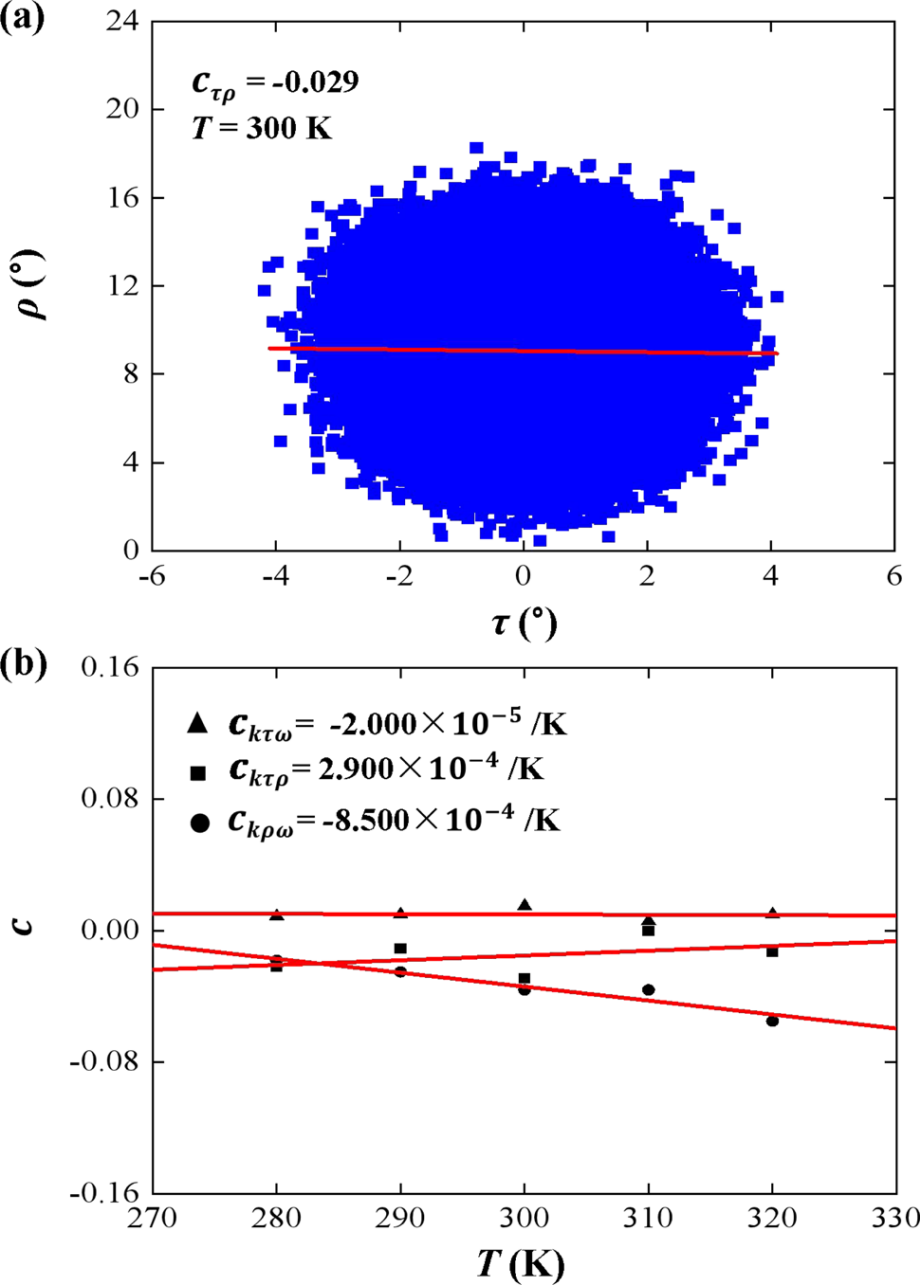
(a) An example of correlation between tilt (*τ*) and roll (*ρ*). The data correction coefficient is *c*_*τρ*_ = −0.029. (b) Temperature dependence of correlation coefficient *c*_*τρ*_, *c*_*τω*_, and *c*_*ρω*_ on temperature *T*. The line is a fitting result with a slope of *c*_*k*_*τρ*__ = 2.900 × 10^−4^ K^−1^, *c*_*k*_*τω*__ = −2.000 × 10^−5^ K^−1^, and *c*_*k*_*ρω*__ = −8.500 × 10^−4^ K^−1^.

**Fig. 5:**
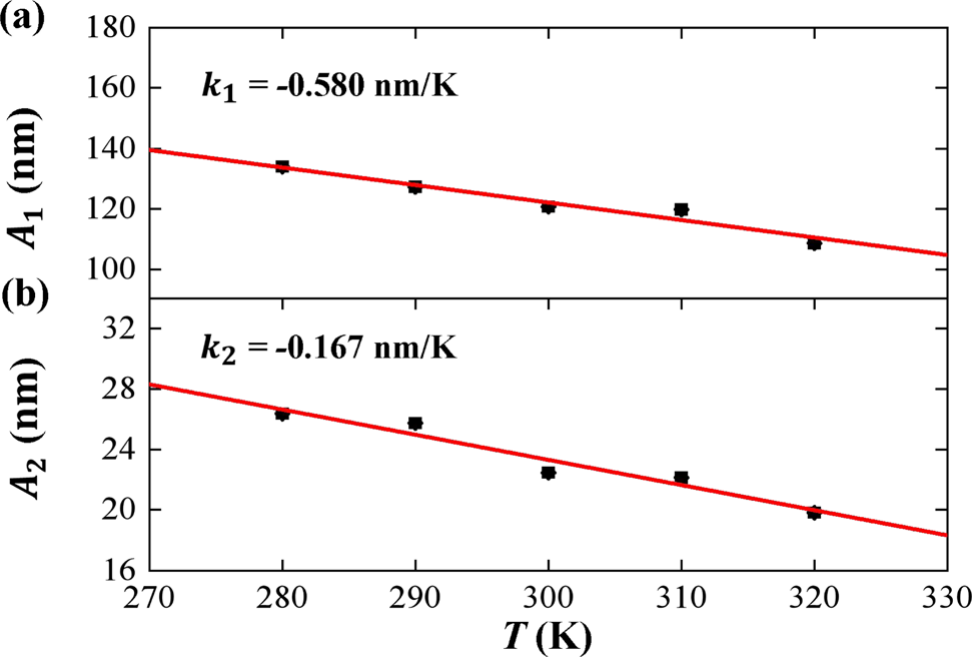
The temperature dependence of tilt stiffness *A*_1_, roll stiffness *A*_2_. (a) The function of tilt stiffness *A*_1_ as a function of temperature *T*, and the line is a fitting result with a slope of *k*_1_ = −0.580 nm K^−1^. (b) The function of roll stiffness *A*_2_ as a function of temperature *T*, and the line is a fitting result with a slope of *k*_2_ = −0.167 nm K^−1^.

**Fig. 6:**
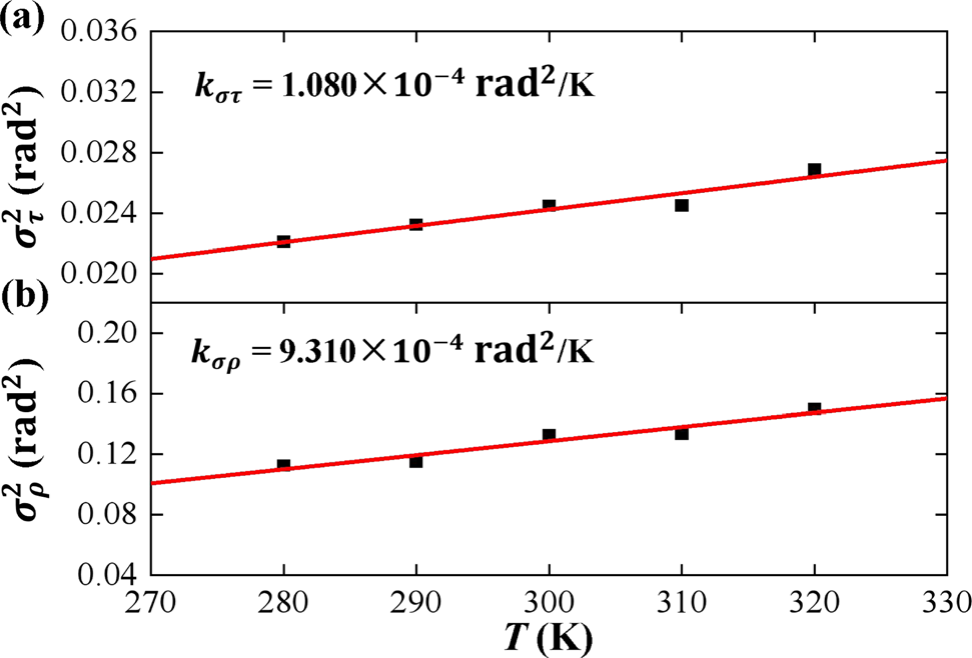
(a) The variance of cumulative tilt *σ*_*τ*_^2^ as a function of temperature *T*. The line is a fitting result with a slope of *k*_*στ*_ = 1.080 × 10^−4^ rad^2^ K^−1^. (b) The variance of cumulative roll *σ*_*ρ*_^2^ as a function of temperature *T*. The line is a fitting result with a slope of *k*_*σρ*_ = 9.310 × 10^−4^ rad^2^ K^−1^.

**Fig. 7:**
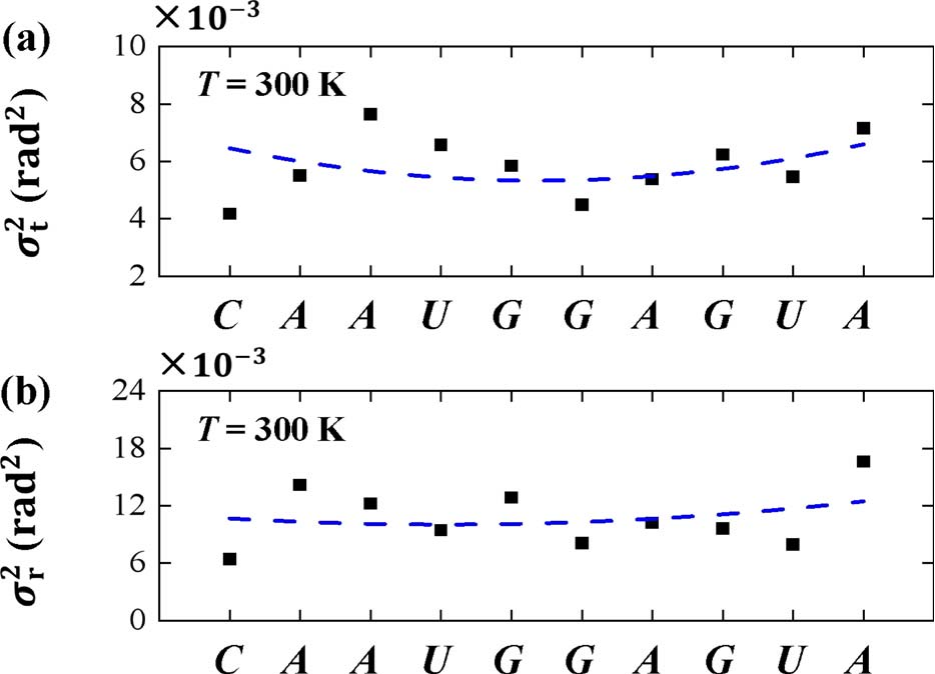
(a) The variance of tilt *σ*_t_^2^ for each bp *T* = 300 K. (b) The variance of roll *σ*_r_^2^ for each bp *T* = 300 K. The dashed curves denote the varying trends along dsRNA sequences.

In this study, we focus on the influence of temperature on the bend elasticity of dsRNA, which is characterized by the bending persistence length based on the WLC model and the bending anisotropy based on the MS model. The temperature was adjusted from *T* = 280 K to *T* = 320 K with steps of Δ*T* = 10 K to demonstrate the temperature-dependent bend elasticity of dsRNA. In Subsection 3.1, we discuss the dependence of bending elasticities in Fig. 2 and 3; we analyzed the temperature dependence of tilt and roll stiffnesses, as well as their bending anisotropy, based on the MS model in Fig. 4–7 in Subsection 3.2.

### 3.1 Temperature-dependent bend elasticity

We plotted the bending persistence length *l*_B_ as a function of temperature *T* in Fig. 2, according to eqn (1) based on the WLC model. In Fig. 2(a), a typical example is shown, where the 
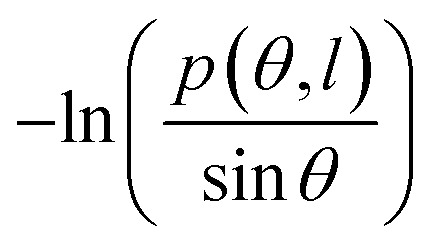
 is plotted as a function of the bending angle *θ* at *T* = 300 K. We used the quadratic curve to fit the MD data and obtained the bending persistence length *l*_B_ = 62.06 ± 0.41 nm at *T* = 300 K. Our MD data are in line with the available all-atom MD simulation data where *l*_B_ ranges from 66 nm to 69 nm.^25,27,28^ Previous experiments have suggested that the dsRNA has a persistence length of approximately *l*_B_ = 57–64 nm under the various salt concentrations.^24–26^ In particular, Abels *et al.* used two single-molecule techniques, the MT and AFM, to measure the bending persistence length of dsRNA at room temperature (*T* = ∼298 K) and obtained *l*_B_ = 63.8 ± 0.7 nm by MT and *l*_B_ = 62 ± 2 nm by AFM.^64^ We listed these data in detail in Table S3 of ESI† where the sequence length and ion concentration were also listed. Actually, the bending persistence length of dsRNA decreases as the concentration of CoHex^3+^ increases^11^ and also has a sequence-dependence.^38^ Thus, the main deviation between the current MD results and experimental data was probably due to the different salt concentrations and sequence lengths used in the current simulation and previous works. For a convenient comparison, we inserted these available data into Fig. 2(b), where the bending persistence lengths were plotted as a function of temperature. Here, we predicted the temperature dependence of bending persistence length *l*_B_, as shown in Fig. 2(b), where the temperature *T* over a range from *T* = 280 K to *T* = 320 K. The bending persistence lengths at other temperatures are also obtained by the same fitting methods as those at *T* = 300 K [see Fig. S2 of ESI†]. We observed a linear relationship between the bending persistence length *l*_B_ and temperature *T*, with the slope of *k*_*l*_ = −0.342 nm K^−1^. Previous experiments have reported the linear dependence of temperature on the bending persistence lengths of dsDNA,^14,17^ and all-atom simulations have also reproduced the linear temperature dependence on the bending persistence lengths of dsDNA where the bending persistence length *l*_B_ decreases as temperature *T* increases, with a slope of −0.29 nm K^−1^.^21^

We used the rotational parameters to understand the temperature dependence of *l*_B_, as shown in Fig. 3. We plotted the bending angle 〈*θ*〉 as function of temperature *T* in Fig. 3(a), where *θ* is bending angle between two successive bps and 〈⋯〉 denotes assemble average. We obtained the 〈*θ*〉 from the MD trajectory data, and 〈*θ*〉 = 9.05° at *T* = 300 K. We note that the average bending angle 〈*θ*〉 is much greater than that of dsDNA.^21,27,62^ Furthermore, we predicted the linear dependence of the average bending angle 〈*θ*〉 on the temperature *T*, as shown in Fig. 3(a). The results showed that the bending angle 〈*θ*〉 increased as the temperature *T* increased, with a slope of *k*_*θ*_ = 0.021° K^−1^, which is similar to the dsDNA case.^21^ We are aware that the smaller bending angle reflects the stronger dsDNA rigidity, in which the upward trend illustrated that the dsRNA chain becomes more flexible with the increasing *T*, supported by the short WLC model about the conformational variations.^58,65^ In the TWLC model, the bending angle is related to two orthogonal angles, the tilt and roll angles, which hold *θ*^2^ = *τ*^2^ + *ρ*^2^.^31,35^ To analyze the bending elasticity in more detail, we plotted the 〈*τ*〉 and 〈*ρ*〉 as functions of temperature *T*, as shown in Fig. 3(b) and (c), where *τ* and *ρ* are the tilt and roll angles, respectively, between two successive bps. We also listed the more detailed data about the tilt and roll angles at various temperatures in Table S1 of ESI.† We obtained 〈*τ*〉 = 0.08° and 〈*ρ*〉 = 9.05° at *T* = 300 K, which are in line with the available data from the various dsRNA sequences,^25,27,28^ for example, 〈*ρ*〉 = 7.5 ± 3.47° and 〈*τ*〉 = −0.01 ± 4.36° for a 16 bps dsRNA sequence used in the previous MD simulation.^28^ The results showed that the average roll angle 〈*ρ*〉 has a linear relationship with temperature *T* on a slope of *k*_*ρ*_ = 0.021° K^−1^. However, the average tilt angle 〈*τ*〉almost maintain unchanged value, with a slope of *k*_*τ*_ = 0.000° K^−1^, as shown in Fig. 3(b) and (c), which suggests that the bending elasticity is mainly derived from the roll angles.^66^

### 3.2 Temperature-dependent tilt and roll stiffnesses

Tilt, roll, and twist are three rotational parameters that play important roles in the bend and twist stiffnesses. In the MS model, the tilt and roll are the two orthogonal components of the bend elasticity. In the current simulations, we concentrated on the bend elasticities involving the tilt and roll angles, as well as the bending anisotropy in Fig. 4 and 5.

First, we analyzed the tilt and roll correlations and showed an example at *T* = 300 K in Fig. 4(a), in which the data between the tilt and roll have negative correlations, with a negative slope of *c*_*τρ*_ = −0.029. This weak correlation indicates that the tilt angle was almost independent of the roll angle at *T* = 300 K. A previous MD simulation also indicated that there was a weak correlation between the tilt and roll angles for dsRNA and dsDNA.^25^ Correlations between tilt and roll are presented in a manner similar to the previous simulations.^43,57^ We also provided the tilt and roll correlations at other temperature in Fig. S3 of ESI,† which indicated the correlations between the tilt and roll are also weak. To demonstrate the temperature-dependence of this correlation clearly, we plotted the correlation coefficient as a function of the temperature *T*, as shown in Fig. 4(b). We then observed a linear relationship between the correlation coefficient *c*_*τρ*_ and temperature *T* with a slope of *c*_*k*_*τρ*__ = 2.900 × 10^−4^ K^−1^. The very weak upward trend indicates that the correlation between the tilt and roll angles is independent of the temperature *T*. Actually, the correlations of tilt and roll to the third angle, twist, cannot be neglected in dsRNA, as shown in Fig. 4(b) [see the tilt–twist correlations and roll–twist correlations at various temperatures in Fig. S4 and S5 of ESI†], which differs from the available data for dsDNA.^25^ This non-correlation between the tilt and roll enables us to analyze the bend elasticity based on the tilt and roll stiffnesses.

We then considered two elastic components, tilt and roll stiffnesses, for the bend elasticity illustrated in the MS model. The MS model has been successfully used in dsDNA to explain the bend and twist elasticities by considering the twist-bending coupling.^31–35^ This is due to undistortion of dsDNA, where the bending angle is relatively small, which leads to the rotational symmetry of tilt angle. The dsRNA is more bent than dsDNA where the average roll between two successive bps is 〈*ρ*〉 = 9.05°. This causes the nonzero terms *K*_*τω*_ ≠ 0 [refer to the **K** elements at various temperatures in Table S2 of ESI†], leading to the deviation of the MS model. However, the *K*_*ρω*_ is much larger than *K*_*τω*_, which enables us to analyze the tilt and roll components for bending elasticities. This is also supported by the fact that the Pearson correlation coefficients between the roll and twist are much larger than those between tilt and twist [refer to Table S2 in ESI† for *K*_*ρω*_ and *K*_*τω*_ at various temperatures]. We obtained the tilt and roll modulus, *K*_*ττ*_ and *K*_*ρρ*_, according to eqn (5) and (6), and plotted the tilt and roll stiffnesses (tilt and roll persistence lengths), *A*_1_ and *A*_2_, as function of temperature *T*, in Fig. 5. Here, the tilt stiffness *A*_1_ is determined by^31,32^7
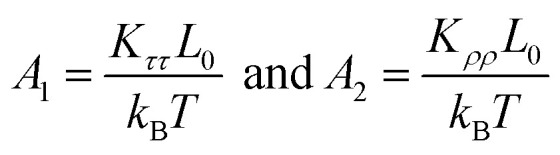
where *L*_0_ denotes the contour length. We obtained *A*_1_ = 120.80 nm by substituting *K*_*ττ*_ = 169.39 pN nm and *A*_2_ = 22.46 nm by substituting *K*_*ρρ*_ = 31.50 pN nm at *T* = 300 K, where *L*_0_ = 2.954 nm. The available data showed that the tilt stiffness *A*_1_ are about ∼100 nm for dsDNA,^31,32,36,37^ whereas the roll stiffness *A*_2_ = 39 nm (ref. 37) and *A*_2_ = 38.8 nm(ref. 32) for dsDNA near the room temperature. Our observations showed that dsRNA has a smaller roll stiffness *A*_2_ than those in dsDNA. It is meaningful to use the asymmetric parameter to describe the bending anisotropy, which is defined as follows^29,30^

8 *B* = (*A*_1_ − *A*_2_)/2

In the current simulations, the dsRNA has an asymmetry parameter of *B* = 49.17 nm, which indicates a much larger bending anisotropic bending elasticity. This asymmetry parameter was much larger than that in dsDNA where the asymmetry parameter was suggested to be *B* = 19 nm.^30^ This anisotropic bending elasticity was also observed in the MD simulations of the dsRNA and dsDNA.^38^ Importantly, our simulation results predicted that the tilt stiffness *A*_1_ and roll stiffness *A*_2_ are temperature-dependent, as shown in Fig. 5(a) and (b), respectively. The data fitting indicated a linear relationship between *A*_1_ and *T* with a slope of *k*_1_ = −0.580 nm K^−1^, while the same is true for the roll angle where *A*_2_ decreases with *T* by a slope of *k*_2_ = −0.167 nm K^−1^. Our observations were supported by a theoretical analysis in which there was a linear relationship between the stiffness matrix **K** and temperature *T*.^16^ We also show the effect of temperature on the anisotropic bending elasticity in Fig. S6.† Our simulation results indicate that asymmetry parameter *B* decreases from 53.85 nm to 44.42 nm when the temperature *T* increases from 280 K to 320 K, with a slope of *k*_3_ = −0.208 nm K^−1^, indicating that the bending anisotropy become weaker as temperature increases.

### 3.3 Thermal fluctuations in tilt and roll angles

According to thermal fluctuations, the elasticity can be described by the covariances between the deformation variables.^22^ Then, we delve into the mechanism governing the temperature dependence of tilt stiffness *A*_1_ and roll stiffness *A*_2_, as shown in Fig. 6. eqn (5) and (6) suggest that the stiffness matrix **K** can be described by the Pearson correlation coefficient cov(*i*, *j*) and thermal fluctuation *σ*_*i*_^2^. This enables us to estimate that the tilt stiffness *A*_1_ and roll stiffness *A*_2_ have approximate forms, similar to the stretch–twist elastic matrix,^21,22^9
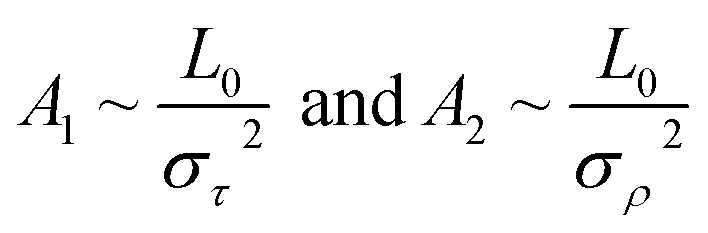


As the contour length *L*_0_ remains unchanged values with temperature *T*, we present the factors *σ*_*τ*_^2^ and *σ*_*ρ*_^2^ as functions of temperature *T* to elucidate the decline in tilt stiffness *A*_1_ and roll stiffness *A*_2_, as shown in Fig. 6. The results indicate that both *σ*_*τ*_^2^ and *σ*_*ρ*_^2^ increase as the temperature *T* increases, displaying nearly linear trends, which is similar to the data obtained from MD simulations of dsDNA.^21^ These findings suggest that the reductions in tilt stiffness *A*_1_ and roll stiffness *A*_2_ are attributed to the thermal fluctuation components *σ*_*τ*_^2^ and *σ*_*ρ*_^2^, respectively. However, the fitting results suggest that the decline rates differ between the *σ*_*τ*_^2^ and *σ*_*ρ*_^2^ as the temperature *T* increases. Specifically, *σ*_*τ*_^2^ increases with a slope of *k*_*στ*_ = 1.080 × 10^−4^ rad^2^ K^−1^ while *k*_*σρ*_ = 9.310 × 10^−4^ rad^2^ K^−1^ for *σ*_*ρ*_^2^. This implies that the thermal fluctuation of the roll angle *σ*_*ρ*_^2^ depends more obviously on the temperature *T* than on the tilt angle, which plays a stronger role in the *T*-dependent roll stiffness *A*_2_.

To understand the thermal fluctuations in more detail, we investigated the thermal fluctuation components *σ*_t_^2^ and *σ*_r_^2^ for each base pair. An example at *T* = 300 K was shown in Figs. 7(a) and (b), respectively. The data showed that *σ*_r_^2^ is greater than *σ*_t_^2^ in each bp, and generally the *σ*_t_^2^ and *σ*_r_^2^ in U and A bps are greater than those in C and G bps, which is in line with the sequence-dependent data.^38,67^ The min values of *σ*_t_^2^ and *σ*_r_^2^ appear in the center of the sequence and the larger values at the two ends, exhibiting the flexibilities of these two ends. These flexibilities are consistent with the flexibility origin of short sequences.^62,68^ Here, we used the sequence-dependent *σ*_t_^2^ and *σ*_r_^2^ to clearly illustrate the anisotropic bending elasticities originating from the thermal fluctuations.

## 4 Conclusions

In the present study, we used all-atom MD simulations to investigate the effects of temperature on the bending elasticity of dsRNA. We chose a short dsRNA sequence of 16 bps to analyze the bending persistence length, tilt, and roll stiffness of the dsRNA. We concentrated on the influences of temperature on bending elasticities and bending anisotropy based on WLC and MS models, and analyzed the mechanism of temperature dependence by thermal fluctuations from the rotational deformation variables.

In the current simulations, we obtained the bending persistence length *l*_B_ = 62.06 ± 0.41 nm for dsRNA at *T* = 300 K, which is in line with the available experimental and MD simulation data. We predicted a linear relationship between the bending persistence length *l*_B_ and temperature *T*, with a slope of *k*_*l*_ = −0.342 nm K^−1^. This linear relationship was similar to that observed for dsDNA. To analyze the bending elasticity in more detail, we investigated the 〈*τ*〉 and 〈*ρ*〉 as functions of temperature *T*, and the results showed that the average roll angle 〈*ρ*〉 has a linear relationship with temperature *T* at a slope of *k*_*ρ*_ = 0.021° K^−1^, while the average tilt angle 〈*τ*〉 remained almost unchanged, with a slope of *k*_*τ*_ = 0.000° K^−1^, which suggests that the bending elasticity is mainly from the roll angles. We then investigated the tilt and roll stiffness as well as its bending anisotropy. We obtained a tilt stiffness *A*_1_ = 120.80 nm and roll stiffness *A*_2_ = 22.46 nm at *T* = 300 K. DsRNA has an asymmetry parameter of *B* = 49.17 nm, which indicates a much larger bending anisotropic bending elasticity than that of dsDNA, where the asymmetry parameter was suggested to be *B* = 19 nm. We predicted that the tilt stiffness *A*_1_ and roll stiffness *A*_2_ decrease linearly with increasing temperature *T* in linear manners, with a slope of *k*_1_ = −0.580 nm K^−1^ for tilt stiffness, while the same is true for the roll angle, where *A*_2_ decreases with *T* by a slope of *k*_2_ = −0.167 nm K^−1^. Our MD simulation results showed that asymmetry parameter *B* decreases from 53.85 nm to 44.42 nm when the temperature *T* increases from 280 K to 320 K, with a slope of *k*_3_ = −0.208 nm K^−1^. This suggests that the bending anisotropy weakened as the temperature increased. Then, we delve into the mechanism governing the temperature dependence of tilt stiffness *A*_1_ and roll stiffness *A*_2_, which suggests that the thermal fluctuation of the roll angle *σ*_*ρ*_^2^ depends more on the temperature *T* than on the tilt angle. As we know, the bending of RNA plays a key role in the transmission of genetic information and organisms survive, as well as the nucleic acid nanostructures involved in functions related to drug delivery. Our all-atom MD simulations provide a deeper understanding of the bending elasticity of dsRNA, which probably has potential applications in these aspects.

## Conflicts of interest

There are no conflicts to declare.

## Supplementary Material

RA-014-D4RA02354D-s001
