# Supplementary material for: Effect of temperature on anisotropic bending elasticity of dsRNA: an all-atom molecular dynamics simulation
Source: RSC Adv. 2024 May 28;14(24):17170–7. doi: 10.1039/d4ra02354d (PMC11130765; doi:10.1039/d4ra02354d)
Supplement: RA-014-D4RA02354D-s001 [file RA-014-D4RA02354D-s001.pdf]

Electronic Supplementary Material (ESI) for RSC Advances.
This journal is © The Royal Society of Chemistry 2024

Supporting Information for

"Effect of temperature on anisotropic bending elasticity of dsRNA: An all-atom molecular dynamics simulation"

Xianghong Wang [1, a)], Tingting Huang[1], Liyun Li[2], Yanliang Xu[1]

[1]School of Sino-German Engineering, Shanghai Technical Institute of Electronics and Information, Shanghai 201411, China
[2]Department of Physics, Wenzhou University, Wenzhou, Zhejiang 325035, China.

a)Authors to whom correspondence should be addressed.
Electronic addresses: wangxianghong@stiei.edu.cn (X.Wang)

Fig. S1

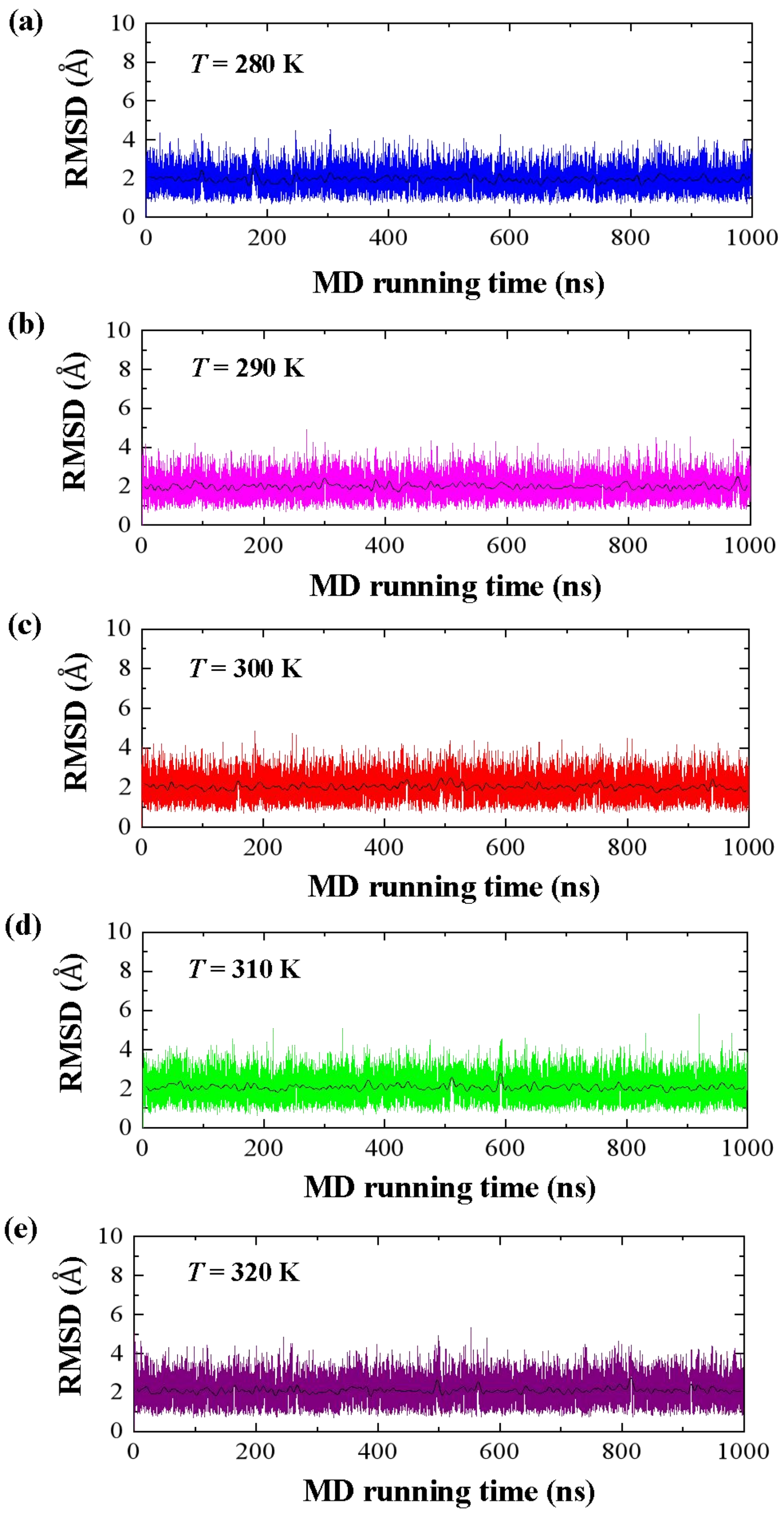

(a)
T = 280 K
RMSD (Å)
MD running time (ns)
(b)
T = 290 K
RMSD (Å)
MD running time (ns)
(c)
T = 300 K
RMSD (Å)
MD running time (ns)
(d)
T = 310 K
RMSD (Å)
MD running time (ns)
(e)
T = 320 K
RMSD (Å)
MD running time (ns)

Fig. S1

(a) Root mean square deviation (RMSD) curves of the 10 bases fragment in the center of the dsRNA at $T = 280$ K, where the black line indicates the average value of the relevant parameter every 2 ns.

(b) Root mean square deviation (RMSD) curves of the 10 bases fragment in the center of the dsRNA at $T = 290$ K, where the black line indicates the average value of the relevant parameter every 2 ns.

(c) Root mean square deviation (RMSD) curves of the 10 bases fragment in the center of the dsRNA at $T = 300$ K, where the black line indicates the average value of the relevant parameter every 2 ns.

(d) Root mean square deviation (RMSD) curves of the 10 bases fragment in the center of the dsRNA at $T = 310$ K, where the black line indicates the average value of the relevant parameter every 2 ns.

(e) Root mean square deviation (RMSD) curves of the 10 bases fragment in the center of the dsRNA at $T = 320$ K, where the black line indicates the average value of the relevant parameter every 2 ns.

Fig. S2

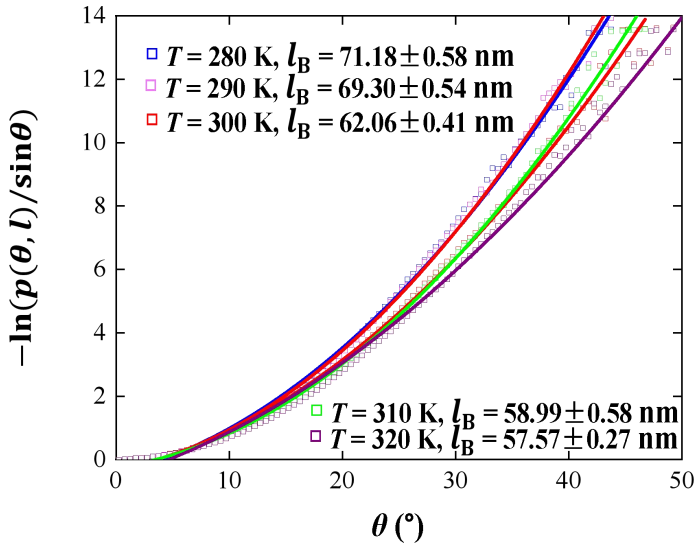

Fig. S2

The relationship between $-\ln(\mathrm{p}(\theta, l)/\sin\theta)$ and bending angle θ at $T = 280\mathrm{K}$, $290\mathrm{K}$, $300\,\mathrm{K}$, $310\mathrm{K}$, $320\mathrm{K}$. The bending angle θ is formed by six consecutive base pairs on each of the 10 base segments at the center of the dsRNA.

Fig. S3

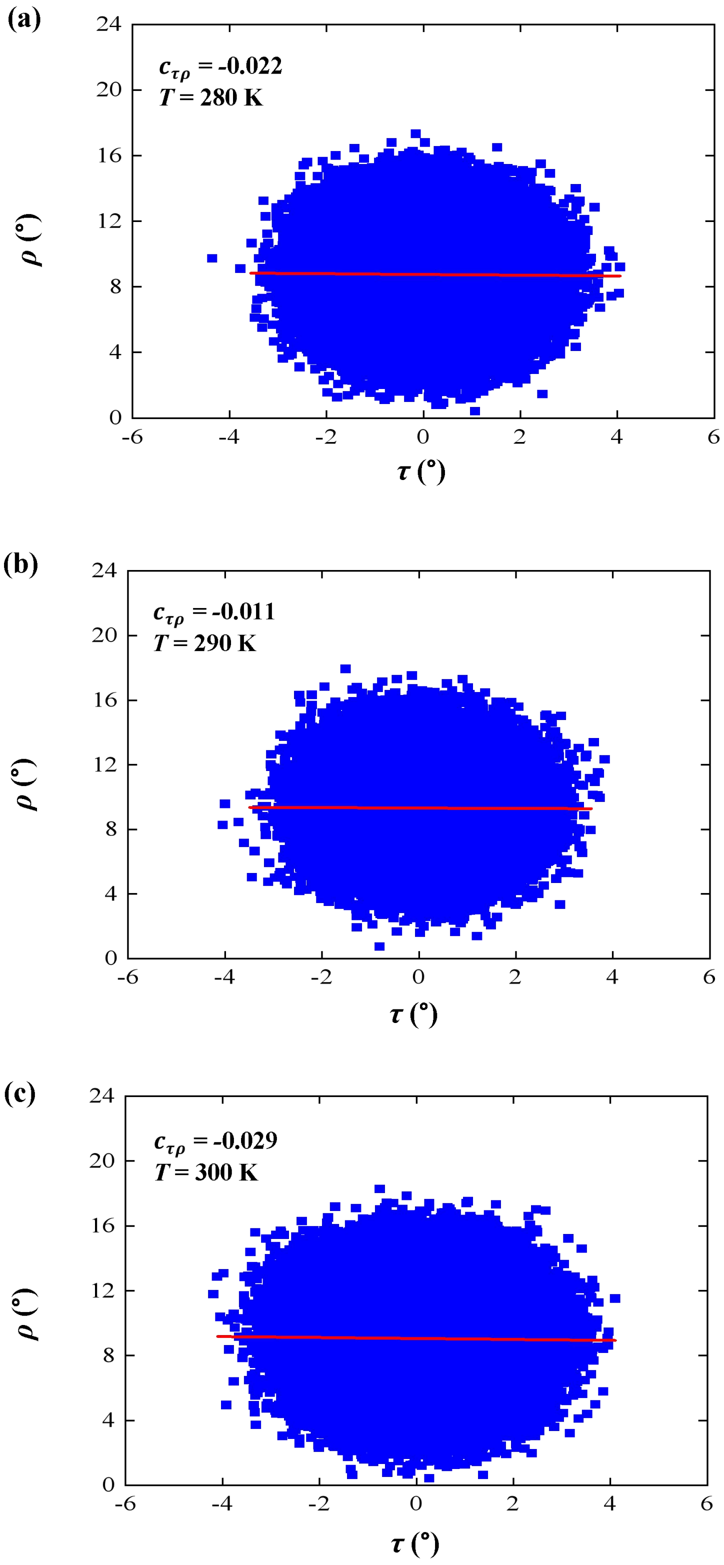

(a)
$c_{\tau\rho}$ = -0.022
T = 280 K
ρ (°)
τ (°)
(b)
$c_{\tau\rho}$ = -0.011
T = 290 K
ρ (°)
τ (°)
(c)
$c_{\tau\rho}$ = -0.029
T = 300 K
ρ (°)
τ (°)

(d)

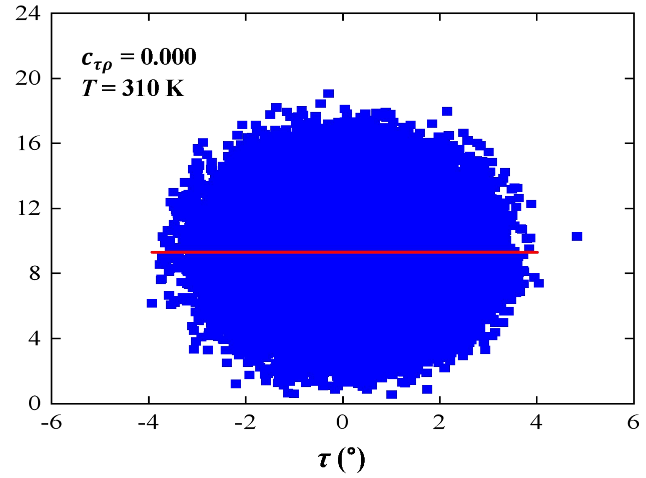

(e)

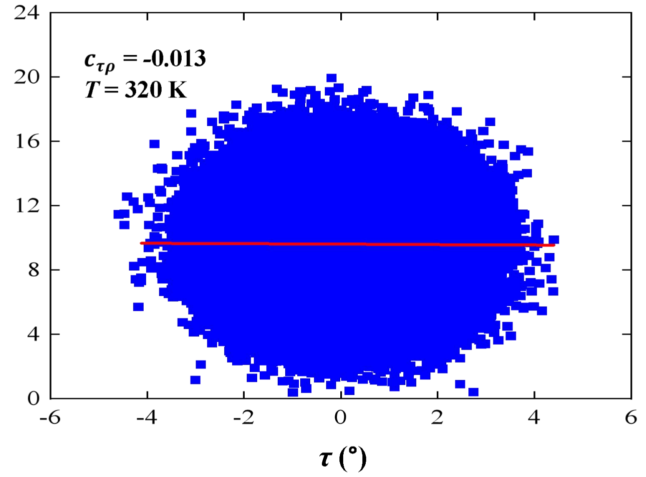

Fig. S3

(a) An example of correlation between tilt (τ) and roll (ρ). The data correlation coefficient is $c_{\tau\rho} =- 0.022$.

(b) An example of correlation between tilt (τ) and roll (ρ). The data correlation coefficient is $c_{\tau\rho} =- 0.011$.

(c) An example of correlation between tilt (τ) and roll (ρ). The data correlation coefficient is $c_{\tau\rho} =- 0.029$.

(d) An example of correlation between tilt (τ) and roll (ρ). The data correlation coefficient is $c_{\tau\rho} = 0.000$.

(e) An example of correlation between tilt (τ) and roll (ρ). The data correlation coefficient is $c_{\tau\rho} =- 0.013$.

Fig. S4

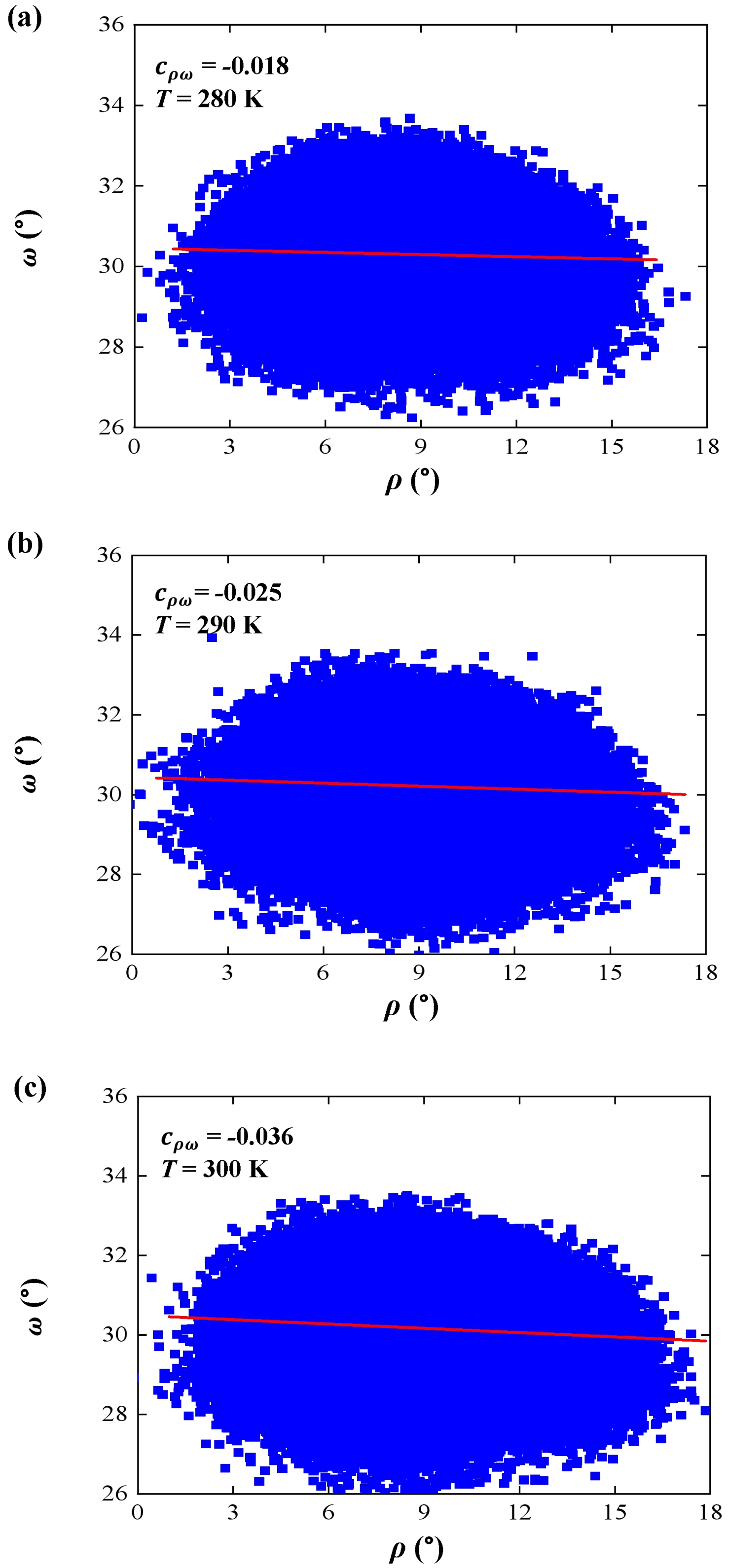
(a)
$c_{\rho\omega}$ = -0.018
T = 280 K
ω (°)
ρ (°)
(b)
$c_{\rho\omega}$= -0.025
T = 290 K
ω (°)
ρ (°)
(c)
$c_{\rho\omega}$ = -0.036
T = 300 K
ω (°)
ρ (°)

(d)

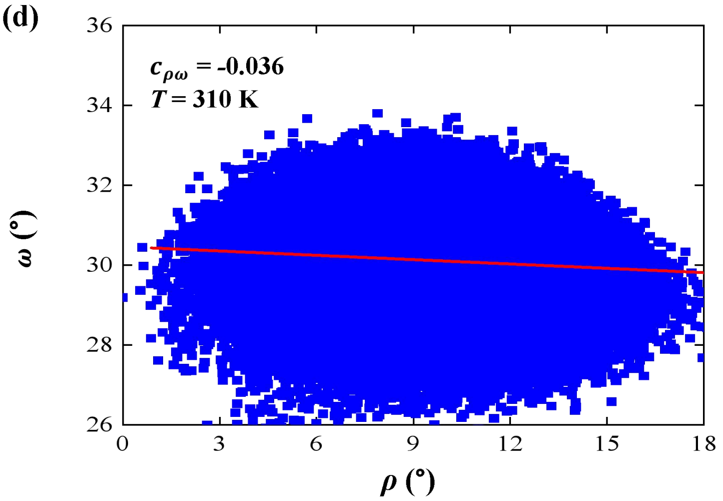

(e)

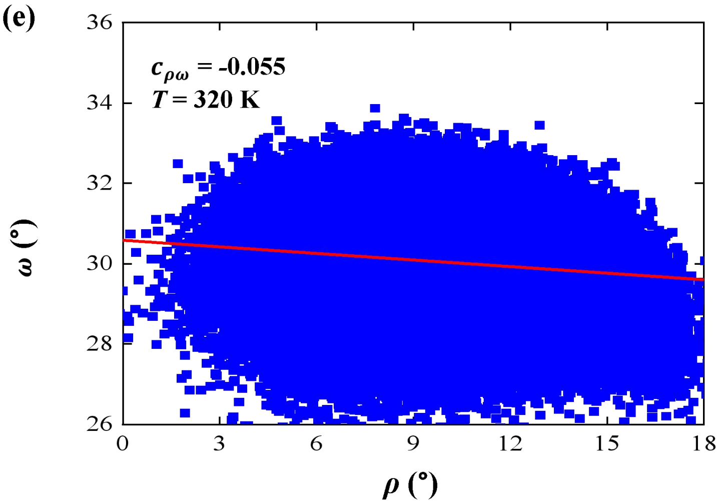

Fig. S4

(a) An example of correlation between roll (ρ) and twist (ω). The data correlation coefficient is $c_{\rho\omega} =- 0.018$.

(b) An example of correlation between roll (ρ) and twist (ω). The data correlation coefficient is $c_{\rho\omega} =- 0.025$.

(c) An example of correlation between roll (ρ) and twist (ω). The data correlation coefficient is $c_{\rho\omega} =- 0.036$.

(d) An example of correlation between roll (ρ) and twist (ω). The data correlation coefficient is $c_{\rho\omega} =- 0.036$.

(e) An example of correlation between roll (ρ) and twist (ω). The data correlation coefficient is $c_{\rho\omega} =- 0.055$.

Fig. S5

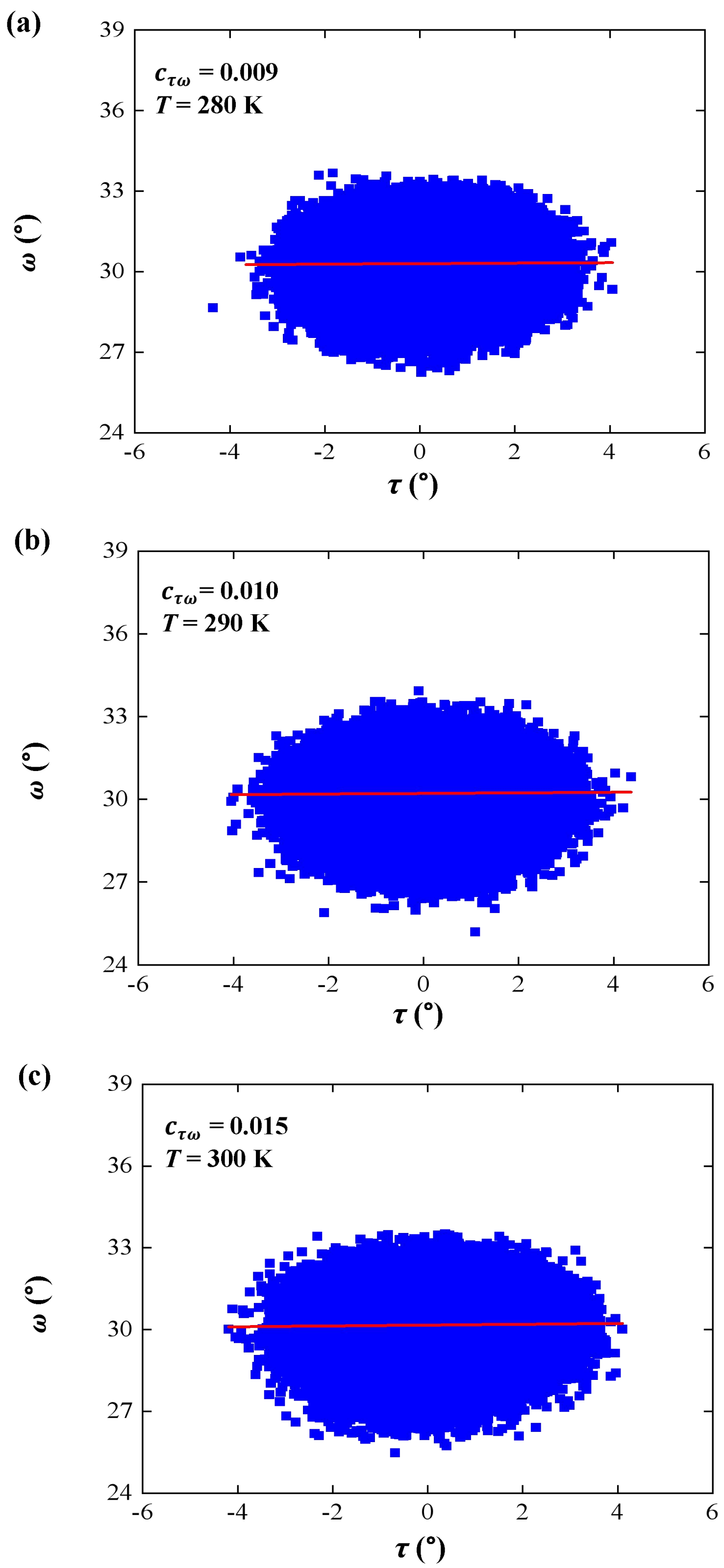

(d)

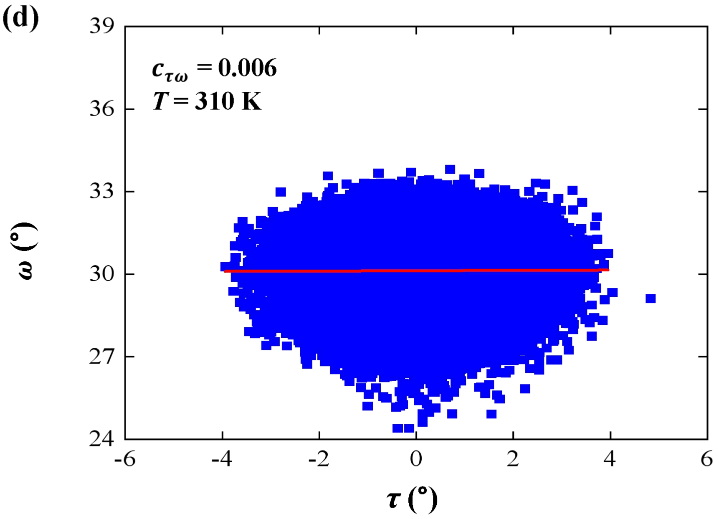

(e)

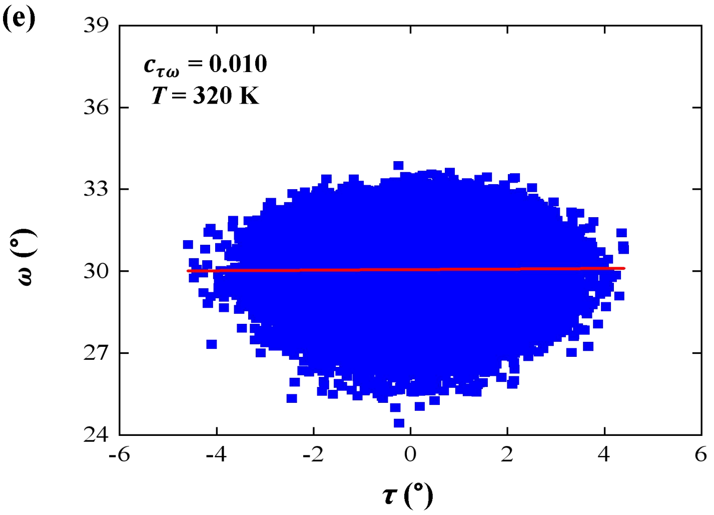

Fig. S5

(a) An example of correlation between tilt (τ) and twist (ω). The data correlation coefficient is $c_{\tau\omega} = 0.009$.

(b) An example of correlation between tilt (τ) and twist (ω). The data correlation coefficient is $c_{\tau\omega} = 0.010$.

(c) An example of correlation between tilt (τ) and twist (ω). The data correlation coefficient is $c_{\tau\omega} = 0.015$.

(d) An example of correlation between tilt (τ) and twist (ω). The data correlation coefficient is $c_{\tau\omega} = 0.006$.

(e) An example of correlation between tilt (τ) and twist (ω). The data correlation coefficient is $c_{\tau\omega} = 0.010$.

Fig. S6

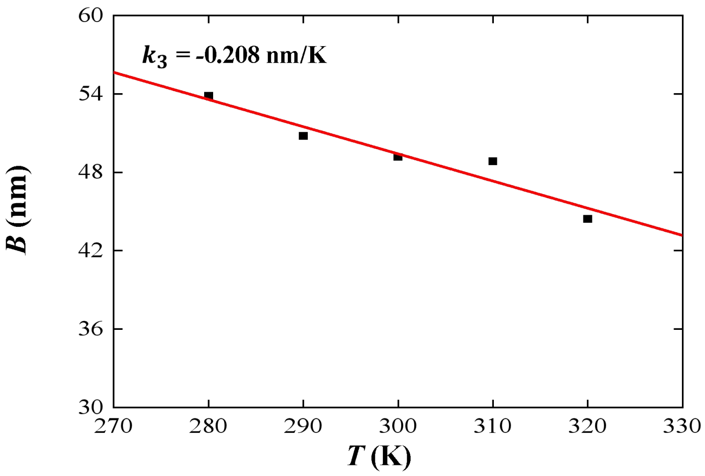

Fig. S6 The function of bending anisotropy B as a function of temperature T, and the line is a fitting result with a slope of $k_3 = -0.208\ \mathrm{nm/K}$.

Table S1

Table S1 The structural parameters and Pearson coefficient of dsRNA at different temperatures

Structure Parameters	280 K	290 K	300 K	310 K	320 K
Tilt, τ (°)	0.10±0.85	0.10±0.87	0.08±0.90	0.10±0.90	0.11±0.94
Roll, ρ (°)	8.75±1.92	8.86±1.95	9.05±2.09	9.30±2.10	9.61±2.22
twist, ω (°)	30.30±0.83	30.22±0.86	30.16±0.88	30.13±0.88	30.06±0.93
corr(τ,ρ)	-0.0199	0.0061	-0.0318	-0.0004	-0.0057
corr(τ,ω)	0.0178	0.0106	0.0065	0.0062	0.0104
corr(ρ,ω)	-0.0409	-0.0567	-0.0857	-0.0860	-0.1309
L_0(nm)	2.9608	2.9575	2.9538	2.9359	2.9190

Table S2

Table S2 Summary of elasticity matrix **K** data for dsRNA at different temperatures.

**K elements**	280 K	290 K	300 K	310 K	320 K
$K_{\tau\tau}$ (pN·nm)	175.04	172.36	169.39	174.68	164.45
$K_{\rho\rho}$ (pN·nm)	34.43	34.86	31.50	32.27	30.00
$K_{\omega\omega}$ (pN·nm)	182.68	179.34	177.96	182.25	172.61
$K_{\tau\rho}$ (pN·nm)	1.49	-0.52	2.29	-0.01	0.31
$K_{\tau\omega}$ (pN·nm)	-3.04	-1.93	-0.67	-1.10	-1.65
$K_{\rho\omega}$ (pN·nm)	3.22	4.49	6.40	6.59	9.42

Table S3

Table S3 Summary of dsRNA elasticity parameters in the previous works.

Subject			Elastic parameters				
Length	Temperature	Ions Concentration	K_{SS}	l_B	K_{TT}	K_{ST}	References
4 kbp	298 K	150 mM NaCl	500±29 pN	60 ± 1 nm			Herrero-Galan et al.[24]
4.2 kbp	298 K	10 mM Tris·HCl and 1mM EDTA	350±100 pN	66.3 nm<br>57± 2 nm	410 ± 8 pN · nm^2	-0.85 ± 0.04 nm	Lipfert et al.[25]
12.5 kbp	295 K	1mM NaCl	630 pN	61 nm			Zhang et al.[26]
16 bp	298.15 K	100 mM NaCl	434±41.09 pN	69 ± 4 nm	214.34 ± 32.48 pN · nm^2		Zhang et al.[27]
16 bp	300 K	Na^+	602.83±27.73 pN	66.99 ± 1.38 nm	416.78 ± 13.16 pN · nm^2		Chhetri et al.[28]
4.2 kbp	298 K	10 mM Tris·HCl and 1mM EDTA		63.8 ± 0.7 nm<br>62 ± 2 nm			Abels et al.[64]
